# Differences in preoperative gastric ultrasound findings in elderly compared to the mid-aged surgical patients: A retrospective observational study

**DOI:** 10.1097/MD.0000000000033595

**Published:** 2023-04-21

**Authors:** Jin Hee Ahn, Jae-Geum Shim, Sung Hyun Lee, Kyoung-Ho Ryu, Mi Yeon Lee, Sinae Kim, Tae-Ryun Gahng, Eun-Ah Cho

**Affiliations:** a Department of Anesthesiology and Pain Medicine, Kangbuk Samsung Hospital, Sungkyunkwan University School of Medicine, Seoul, Republic of Korea; b Division of Biostatistics, Department of R&D Management, Kangbuk Samsung Hospital, Sungkyunkwan University School of Medicine, Seoul, Republic of Korea; c Division of Biostatistics, Biostatics Collaboration Team, Research Core Center, National Cancer Center, Gyeonggi-do, Republic of Korea.

**Keywords:** elderly, gastric ultrasound, old age, preoperative, pulmonary aspiration

## Abstract

This study aimed to compare gastric ultrasound assessments between young and elderly patients, to determine whether the cross-section area (CSA) cutoff values for elderly and young patients should be different, and to suggest CSA cutoff values for elderly patients. This study evaluated the data of 120 patients who underwent elective surgery under general anesthesia between July 2019 and August 2020. Demographic and gastric ultrasound assessment data were retrieved. Patients were divided into the elderly group (n = 58, age: ≥65 years) and young group (n = 62, age: <65 years). The CSAs in the supine and right lateral decubitus positions (RLDP), semiquantitative 3-point Perlas grade (grades 0, 1, and 2), and gastric volume (GV) were determined. CSAs according to different Perlas grades were compared between the 2 groups. To compare normally and non-normally distributed continuous data, Student *t* test and the Mann–Whitney *U* test were used, respectively. Categorical data were compared using the chi-square test or Fisher exact test, as appropriate. The receiver operating characteristic (ROC) curves were built for the CSAs to predict pulmonary aspiration. The CSA cutoff values for predicting a high risk of pulmonary aspiration in both the groups were determined. Among patients with Perlas grade 0, the CSA_supine_ (*P* = .002) and CSA_RLDP_ (*P* = .002) were greater in the elderly group than in the young group. The specificity, positive predictive value, and accuracy of the CSA decreased when the CSA cutoff value for the young group was applied to the elderly group. The CSA cutoff values for the elderly group were: CSA_supine_, 6.92 cm^2^ and CSA_RLDP_, 10.65 cm^2^. The CSA of the empty stomach was greater in elderly patients than in young patients. We suggest that the following CSA cutoff values should be used for predicting pulmonary aspiration risk in elderly patients: CSA_supine_, 6.92 cm^2^ and CSA_RLDP_, 10.65 cm^2^.

## 1. Introduction

Gastric ultrasound is a noninvasive and reliable preoperative point-of-care tool for assessing the risk of pulmonary aspiration.^[1–5]^ The assessment of pulmonary aspiration risk with gastric ultrasound involves qualitative and quantitative evaluation of residual gastric contents. In qualitative assessment, the gastric antrum is scanned for its emptiness, that is, whether it is completely empty (collapsed appearance) or contains fluids (hypoechoic and round appearance) or solids (hyperechoic or heterogeneous echogenicity).^[4,6,7]^ Based on the findings of qualitative assessment, the gastric antrum is classified using a semi-quantitative 3-point grading scale, the Perlas grading scale (0 = empty; 1 = low to slightly increased gastric volume [GV]; and 2 = increased GV).^[1,4,6,8]^ In quantitative assessment, the residual GV is estimated by measuring the antral cross-sectional area (CSA) and substituting the CSA into a mathematical formula.^[4]^ Alternatively, the CSA cutoff value can be used as a diagnostic indicator of a high risk of pulmonary aspiration.^[9]^

While it is known that gastric function is well preserved in the elderly patients, the relationship of aging and gastric emptying is inconclusive.^[10,11]^ While there are some debates, gastric emptying can be delayed in elderly patients due to underlying diseases, polypharmacy, and undiagnosed gastroesophageal reflux.^[11–13]^ Therefore, it seems even more important to assess gastric emptying with gastric ultrasound in the elderly patients. As with gastric ultrasound, age is a major factor for calculation of GV.^[4,10,14]^ Alternatively, the relationship between the CSA and residual GV in elderly patients is different from that in younger patients.^[14]^ Thus, for a given GV, the CSA is greater in older patients than in their younger counterpart, due to difference in the gastric compliance.^[14]^ Therefore, using the same CSA cutoff values for both groups may lead to overdiagnosis of delayed gastric emptying in elderly patients.

Although there is some research on the CSA cutoff values for assessing gastric emptying, most studies have been mainly conducted in the middle-aged patients.^[9,15,16]^ Therefore, we designed our study to determine the appropriate cutoff values for the elderly group. Our study evaluated and compared the outcomes of gastric ultrasound in elderly and young patients undergoing elective surgery. This retrospective observational study aimed to: compare the CSA between young and elderly patients according to the Perlas grade; assess whether different CSA cutoff values are required for the elderly patients and the young patients; and suggest the CSA cutoff values for pulmonary aspiration risk in the young and elderly patients.

## 2. Methods

We conducted a retrospective observational study. This study was approved by the Institutional Ethics Board of Kangbuk Samsung Hospital, Seoul, Korea (Institutional Review Board number: 2021-08-006), and we confirm that all experiments were performed in accordance with relevant guidelines and regulations. This study used only anonymized data and hence, the requirement for consent to participate was waived by the Institutional Ethics Board of Kangbuk Samsung Hospital, Seoul, Korea. We followed the STROBE (Strengthening the Reporting of Observational Studies in Epidemiology) statement.

Data of patients who underwent elective surgery under general anesthesia between July 2019 and August 2020 were retrospectively collected. Inclusion criteria were as follows: age more than 18 years and ASA (American Society of Anesthesiologists) class I to III. Exclusion criteria were as follows: pregnancy; alcoholism; drug abuse; underlying diseases that might affect gastric emptying, such as known gastroesophageal reflux disease, ileus, enteral feeding, hiatal hernia, and psychiatric or mental disorders; and no preoperative gastric ultrasound assessment. The patients followed the standard preoperative fasting policy of our hospital: overnight fasting of solid food starting from midnight and allowing clear fluid intake until 2 hours before surgery.

Ultrasound assessments were performed in the waiting area by 2 anesthesiologists (experience of more than 100 gastric ultrasound examinations) using a GE ultrasound system (LogiQ E, GE Healthcare, Piscataway, NJ) and a curved-array transducer (1.6–4.6 MHz) with standard abdominal settings. Gastric ultrasound was performed in 2 positions: the supine position with the head of the bed elevated 45 degrees and the right lateral decubitus positions (RLDP). The antrum was scanned in the epigastrium, located between the left lobe of the liver and pancreas, as previously described.^[17]^ First, the gastric antrum was assessed for emptiness. A gastric antrum without any gastric contents was defined as “empty.” A gastric antrum with clear hypoechoic round appearance on ultrasound was defined as “fluid.” A gastric antrum with hyper- or heterogeneous echogenicity because of the presence of any solid contents was defined as “solid.” The gastric antrum was graded using a semi-quantitative 3-point grading scale, the Perlas grading scale: grade 0, fluid or solid not observed in both the supine position and RLDP; grade 1, fluid observed only in the RLDP; and grade 2, fluid observed in both the supine position and RLDP.^[15]^ Perlas grade 2 suggests that 75% of patients would have a residual GV of >100 mL.^[4]^ In the quantitative assessment, the longest diameter (LD) and the shortest diameter were measured, including the outermost serosa layer, between gastric peristaltic movement. The antral CSA was calculated using the following formula: CSA=ϕ×LD×SD/4.^[17]^ Estimated GV was calculated using the formula reported by Perlas et al^[14]^; GV=27.0 + 14.6×CSA _RDLP_−1.28×age.

The risk of pulmonary aspiration was assessed based on the classification described in a previous study.^[4]^ High pulmonary aspiration risk was defined by the presence of solid contents in the stomach or a residual GV per kg of >1.5 mL/kg.^[4,18]^

### 2.1. Statistical analysis

Data are presented as mean ± standard deviation, median (interquartile range), or numbers (percentage), as appropriate. The study subjects were allocated into 2 different groups based on the age: the young (age < 65 years) and elderly (age ≥ 65 years) groups. The continuous variables were tested for normal distribution using the Shapiro–Wilk test. The normally distributed continuous data including age, height, body mass index, and CSA were compared using Student *t* test. Mann–Whitney *U* test was used to compare non-normally distributed data (ASA physical class, fasting hours for solids and fluids, and Perlas grade). Categorical data (sex, hypertension, diabetes mellitus, chronic kidney disease, thyroid disease, coronary artery disease, and qualitative assessment gastric ultrasound data) were compared using the chi-square test or Fisher exact test, as appropriate. A *P* value of <.05 was considered statistically significant.

To test the discriminating ability of CSA_supine_ and CSA _RLDP_ for the prediction of high pulmonary aspiration risk in each group, the receiver operating characteristic (ROC) curve was created, and area under the ROC curve (AUC) was calculated and compared with the Hanley-McNeil test (AUC = 0.5, no prediction possible; AUC = 1.0, best possible prediction). The cutoff values of CSA_supine_ and CSA _RLDP_ for predicting high pulmonary aspiration risk were the maximum values of the Youden index [sensitivity + (specificity–1)]. The cutoff values of the young and elderly groups were compared by applying the CSA cutoff values of the young group to the elderly group, and vice versa. SPSS Statistics for Windows (version 24.0; IBM Corp., Armonk, New York) was used for statistical analyses.

## 3. Results

**Figure 1.**
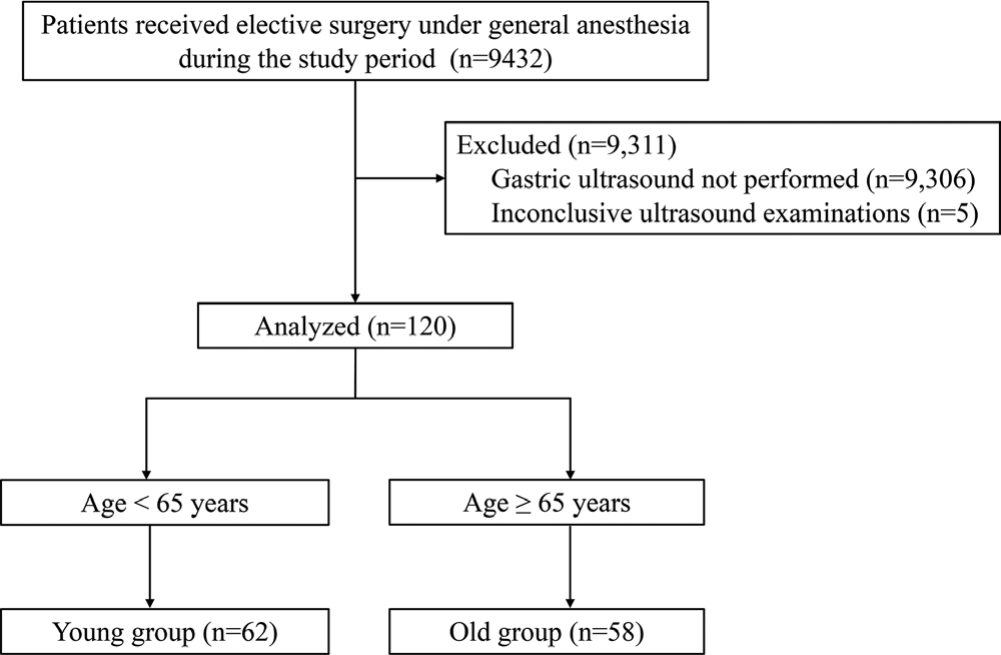
Patient flow diagram.

A total of 9432 patients underwent elective surgery under general anesthesia during the study period, of which 9311 patients were excluded from the study population (including 9306 patients who did not receive preoperative gastric ultrasound and 5 patients with inconclusive ultrasound examinations). Thus, 120 patients were included in this study. The patients were divided into 2 groups: young group, 62 patients and old group, 58 patients (Fig. 1).

The demographic characteristics of the patients are described in Table 1. The average ages of the young and elderly groups were 39 ± 10.52 and 74 ± 5.56 years, respectively (*P* < .001). The proportions of females in the young and old groups were 100% and 20.1%, respectively (*P* < .001). Body mass index in the young group was 22.1 ± 3.03 kg/m^2^ and that in the elderly group was 25.0 ± 2.91 kg/m^2^ (*P* < .001). The ASA class was higher in the elderly group (2 [1–2]) than in the young group (1 [1–1], <0.001). Fasting time for solids in the young group was 12 (10–15) hours and that in the elderly group was 15 (14–19) hours (*P* < .001). Fasting time for fluids did not differ between the groups (*P* > .05).

**Table 1:** Demographic data of the study population.

	Overall (n = 120)	Young group (n = 62)	Elderly group (n = 58)	*p* value
Age, yr	56 ± 19	39 ± 1	74 ± 1	<.001*
Sex, female	74 (61.7)	62 (100)	12 (20.1)	<.001*
Weight, kg	61.9 ± 10.4	57.4 ± 1.0	66.8 ± 1.4	<.001*
Height, cm	162.2 ± 6.6	161.3 ± 0.7	163.1 ± 0.99	.154
Body mass index, kg/m^2^	23.5 ± 3.3	22.1 ± 0.4	25.0 ± 0.4	<.001*
ASA physical class				<.001*
1	62 (51.67)	52 (83.87)	16 (27.59)	
2	58 (48.33)	10 (16.13)	42 (72.41)	
Hypertension	30 (2.5)	0 (0)	30 (51.7)	<.001*
Diabetes mellitus	5 (4.2)	1 (1.6)	4 (6.9)	.196
Chronic kidney disease	4 (3.3)	0 (0)	4 (6.9)	.052
Thyroid disease	5 (4.2)	3 (4.8)	2 (3.4)	>.999
Coronary artery disease	4 (3.3)	0 (0)	4 (6.9)	.052
Fasting hr for solids, hr	14 (12–16)	12 (10–15)	15 (14–19)	<.001*
Fasting hr for fluids, hr	3 (2–12)	3 (2–11)	4 (2–12)	.292

Data are presented as mean ± standard deviation, *n* (%) or median (interquartile range).

ASA = American Society of Anesthesiologists.

* *P* < .05.

The gastric ultrasound findings are presented in Table 2. The qualitative assessments revealed that the proportion of the empty (30 [48.4%] in the young group vs 37 [63.8%] in the elderly group) and liquid (32 [51.6%] in the young group vs 21 [36.2%] in the elderly group) did not differ between the 2 groups. In addition, no solid contents were found in both the groups (*P* = .089). The Perlas grade did not differ between the young group (grade 1 [0–1]) and the elderly group (grade 0 [0–1], *P* = .202). The CSA_supine_ and CSA_right lateral decubitus position (RLDP)_ were compared between the young and elderly groups according to the different Perlas grades. Among patients with Perlas grade 0, both the CSA_supine_ (*P* = .005) and CSA _RLDP_ (*P* = .002) were greater in the elderly group (CSA_supine_ 5.12 ± 1.99 cm^2^, CSA _RLDP_ 6.24 ± 2.62 cm^2^) than in the young group (CSA_supine_ 3.92 ± 1.04 cm^2^, and CSA _RLDP_ 4.58 ± 1.17 cm^2^). Among patients with Perlas grade 1, the CSA_supine_ was greater in the elderly group (5.32 ± 2.85 cm^2^) than the young group (3.49 ± 0.73 cm^2^, *P* < .05; however, the CSA _RLDP_ (*P* = .324) did not differ between the 2 groups. Among patients with Perlas grade 2, the CSA_supine_ (*P* = .353) and CSA _RLDP_ (*P* = .749) did not differ between the 2 groups.

**Table 2:** Gastric ultrasound assessments (qualitative assessment, semi-quantitative 3-point Perlas grading, and cross-sectional area (CSA) according to each Perlas grade).

	Young group (n = 62)	Elderly group (n = 58)	*P* value
Empty/liquid/solid	30/32/0 (48.4/51.6/0)	37/21/0 (63.8/36.2/0)	.089
Perlas grade			.131
0	30 (48.4)	32 (63.8)	
1	23 (37.1)	12 (20.7)	
2	9 (14.5)	9 (15.5)	
CSA according to Perlas grade, cm^2^			
Perlas 0	(n = 30)	(n = 37)	
CSA_supine_	3.92 ± 1.04	5.12 ± 1.99	.005*
CSA_RLDP_	4.58 ± 1.17	6.24 ± 2.62	.002*
Perlas 1	(n = 23)	(n = 12)	
CSA_supine_	3.49 ± 0.73	5.32 ± 2.85	.006*
CSA_RLDP_	7.27 ± 2.26	8.06 ± 2.12	.324
Perlas 2	(n = 9)	(n = 9)	
CSA_supine_	5.95 ± 0.98	7.08 ± 3.33	.353
CSA_RLDP_	9.59 ± 5.68	10.38 ± 4.57	.749

Data are presented as *n* (%), median (interquartile range) or mean ± standard deviation.

CSA = cross-sectional area, RLDP = right lateral decubitus position.

* *P* < .05.

**Figure 2.**
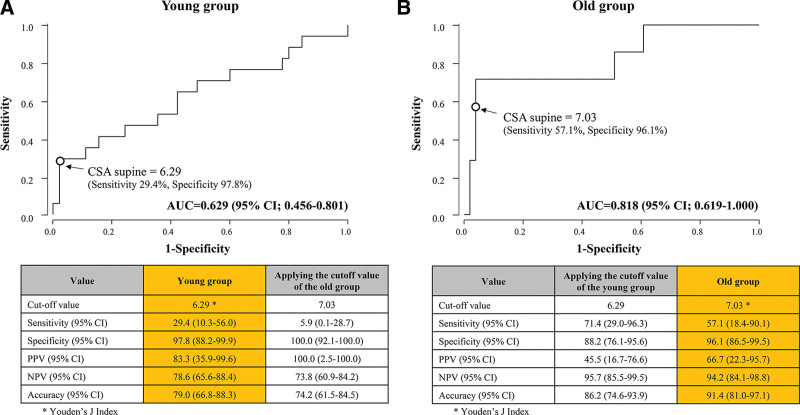
Analysis of ROC curve of the CSA measured in the supine position for predicting the risk of pulmonary aspiration (defined by a GV per weight of >1.5 mL/kg) in the young group (A) and old group (B). The AUC of CSA_supine_ in the young group was 0.629 (95% CI: 0.456–0.801). The AUC of CSA_supine_ in old group was 0.818 (95% CI: 0.619–1.000). The optimal threshold of CSA_supine_ for identifying patients with a GV per kg of >1.5 mL/kg in the young group was 6.29 cm^2^ (sensitivity: 29.4%; specificity: 97.8%) and that in the old group was 7.03 cm^2^ (sensitivity: 57.1%; specificity: 96.1%). The tables below the graphs show the diagnostic performance, when the cutoff values are applied. The values in the colored columns are the diagnostic values when the CSA cutoff value of each group was applied. The values in the non-colored columns are the diagnostic values when the CSA cutoff value of the counterparts was applied. AUC = area under the receiver operating characteristic curve, CI = confidence interval, CSA = cross-sectional area, GV = gastric volume, NPV = negative predictive value, PPV = positive predictive value, ROC = receiver operating characteristic.

**Figure 3.**
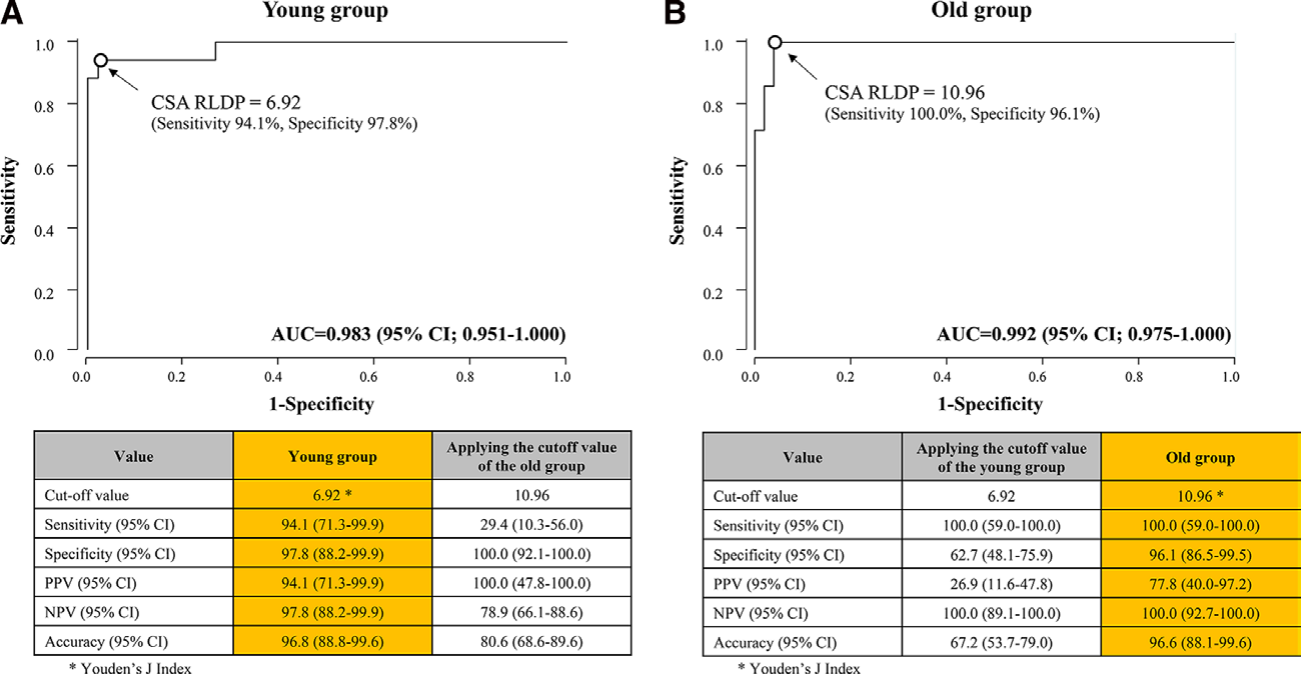
Analysis of ROC curve of the CSA measured in the RLDP for predicting the risk of pulmonary aspiration (defined by a GV per weight of >1.5 mL/kg) in the young group (A) and old group (B). The AUC of CSA _RLDP_ in the young group was 0.983 (95% CI: 0.951–1.000). The AUC CSA _RLDP_ in old group was 0.992 of (95% CI: 0.975–1.000). The optimal threshold of CSA _RLDP_ for discriminating patients with a GV per kg of >1.5 mL/kg in the young group was 6.92 cm^2^ (sensitivity, 94.1%; specificity, 97.8%) and that in the old group was 10.96 cm^2^ (sensitivity, 100%; specificity, 96.1%). The tables below the graphs show the diagnostic values, when the cutoff value of the counterparts was applied. AUC = area under the receiver operating characteristic curve, CI = confidence interval, CSA = cross-sectional area, GV = gastric volume, NPV = negative predictive value, PPV = positive predictive value, RLDP = right lateral decubitus position, ROC = receiver operating characteristic.

The diagnostic performance of Perlas grade 2 for high pulmonary aspiration risk in the elderly and young groups is presented in Table 3. The ROC curves of CSA_supine_ and CSA _RLDP_ for predicting the risk of pulmonary aspiration, defined by a GV per kg of >1.5 mL/kg in the young and old groups are presented in Figures 2 and 3. The CSA_supine_ could not predict high pulmonary aspiration risk in the young group, unlike in the elderly group (young group: area under the ROC curve [AUC], 0.629 [95% confidence interval (CI) 0.456–0.801] and elderly group: AUC, 0.818 [95% CI, 0.619–1.000]). The CSA_supine_ cutoff values for discriminating patients with a GV per kg of >1.5 mL/kg in the young and elderly groups were 6.29 cm^2^ (sensitivity, 29.4%; specificity, 97.8%) and 7.03 cm^2^ (sensitivity, 57.1%; specificity, 96.1%), respectively (Fig. 2). However, the CSA _RLDP_ could predict the risk of pulmonary aspiration in both the groups. The AUCs of CSA _RLDP_ in the young and elderly groups were 0.983 (95% CI: 0.951–1.000) and 0.992 (95% CI: 0.975–1.000), respectively. The CSA _RLDP_ cufoff values for discriminating patients with a GV per kg of >1.5 mL/kg in the young and elderly groups were 6.92 cm^2^ (sensitivity, 94.1%; specificity, 97.8%) and 10.96 cm^2^ (sensitivity, 100%; specificity, 96.1%), respectively. Further, the application of the CSA _RLDP_ cutoff value for the young group to the old group decreased the specificity, positive predictive value, and negative predictive value of CSA _RLDP_ in the old group (Fig. 3).

**Table 3:** Performance of the Perlas grade 2 for the prediction of high pulmonary aspiration risk defined by a residual gastric volume per kilogram of more than 1.5 mL/kg.

	Sensitivity (95% CI)	Specificity (95% CI)	PPV (95% CI)	NPV (95% CI)	Accuracy (95% CI)
Elderly group	94.1 (71.3–99.9)	64.4 (48.8–78.1)	50.0 (31.9–68.1)	96.7 (82.8–99.9)	72.6 (59.8–83.1)
Young group	57.1 (18.4–90.1)	66.7 (52.1–79.2)	19.0 (5.4–41.9)	91.9 (78.1–98.3)	65.5 (51.9–77.5)

CI = confidence interval, NPV = negative predictive value, PPV = positive predictive value.

## 4. Discussion

In this retrospective observational study, we investigated the difference in gastric ultrasound findings between elderly patients and young patients. Among patients with Perlas grade 0, which refers to the empty stomach, the CSA_supine_ and CSA _RLDP_ were greater in the elderly group than in the young group. The CSA_supine_ could not predict the risk of pulmonary aspiration in the young group. However, the CSA _RLDP_ in the young group and the CSA supine and CSA _RLDP_ in the old group could predict the risk of pulmonary aspiration. The CSA _RLDP_ cutoff value for predicting high risk of pulmonary aspiration in the young group was 6.92 cm^2^. In the elderly group, the CSA_supine_ and CSA _RLDP_ cutoff values for predicting high risk of pulmonary aspiration were 7.03 cm^2^ and 10.96 cm^2^, respectively.

Aspiration pneumonia is one of the major complications related to general anesthesia.^[19–21]^ Therefore, it is essential for patients to fast prior to surgery to prevent pulmonary aspiration.^[20,22]^ Nowadays, the emptiness of the stomach can be easily assessed with gastric ultrasound at the bedside.^[23]^ The assessment of pulmonary aspiration risk with gastric ultrasound involves several steps.^[4]^ First, qualitative assessment of gastric contents (i.e., whether the stomach is empty or contains fluid or solid) is performed.^[16]^ The risk of pulmonary aspiration is considered high if solids are present in the stomach and is considered low if the stomach is empty. However, if liquid is present in the stomach, additional quantitative evaluation is required.^[4]^ The risk of pulmonary aspiration is evaluated using a semi-quantitative 3-point grading scale (grades 0, 1, and 2) that considers the difference in qualitative assessments performed in 2 different positions (supine position and RLDP).^[4,15]^ In the quantitative assessment, the CSA cutoff value is used to discriminate patients with a high risk of pulmonary aspiration. Alternatively, the CSA is used to calculate the estimated GV; an estimated GV per kg of >1.5 mL/kg is considered an indicator of high pulmonary aspiration risk.^[4,6]^

Although the mathematical models for calculating GVs are known to be reliable,^[2,3]^ they sometimes lack reproducibility after adjusting for different patient populations.^[17]^ In addition, the assessment of the risk of pulmonary aspiration with mathematical models may be somewhat cumbersome compared with that with the grading system or the CSA. This is because mathematical models involve calculation by combining specific constants. Therefore, we thought that simple and intuitive evaluation methods such as grading and use of CSA cutoff values can be more easily adopted in actual clinical practice. However, because elderly patients have higher gastric compliance than younger patients,^[14]^ we expected that the prediction of pulmonary aspiration risk with a semi-quantitative CSA cutoff value would be different in elderly and young patients.

In our study, we investigated whether Perlas grade 2 predicted a high risk of pulmonary aspiration in each group. The positive predictive value of Perlas grade 2 was low in both the groups, with 50% in the elderly group and 19% in the young group. Based on our study results, we agree with a previous study suggesting that Perlas grade 2 should be used as a screening test and a guide for further pulmonary aspiration risk assessments.^[4]^ However, because our sample size was small, we believe that our results should be validated in well-designed prospective studies with larger sample sizes.

Some studies have reported the use of CSA cutoff values for the diagnosis of a high risk of pulmonary aspiration.^[8,9]^ For example, Bouvet et al reported that a CSA of >320 mm^2^ is associated with an increased risk of pulmonary aspiration.^[8]^ Another study reported that a CSA of 340 mm^2^ indicated an increased risk of pulmonary aspiration with a sensitivity of 91% and a specificity of 71%. The AUC for the diagnosis of a high risk of pulmonary aspiration was 90%.^[9]^ However, the CSA cutoff values presented in these studies have a limitation in that age was not considered. In our study, the CSA cutoff value was greater in elderly patients than in young patients. We believe that this was because of the higher gastric compliance in elderly patients, as revealed in a previous study.^[14]^ Therefore, we believe that the CSA cutoff value for evaluating the risk of pulmonary aspiration should be greater for elderly patients than for young patients. Moreover, the application of the CSA cutoff value for the young group (CSA _RLDP_ = 6.92 cm^2^) to the elderly group decreased the specificity, positive predictive value, and accuracy. Thus, we suggest the following CSA cutoff values for the elderly patients: CSA_supine_, 6.92 cm^2^ and CSA _RLDP_, 10.65 cm^2^.

It is well-known that the accuracy of the assessment of gastric residual liquid with ultrasound in the RLDP is greater than in the supine position because of gravity.^[6]^ However, the assessment cannot be performed in the RLDP in some patients with clinical conditions such as extreme pain hip fracture,^[24]^ mental disorder, and poor cooperation. In this study, the CSA_supine_ had a good predictive value (AUC: 0.818) for high pulmonary aspiration risk. Therefore, we carefully suggest that gastric ultrasound in the supine position with a head up tilt at 45 degrees would be helpful for predicting the risk of pulmonary aspiration in elderly patients, when gastric ultrasound in the RLDP cannot be performed.

This study had some limitations. First, this was a retrospective study. Because the number of patients who underwent preoperative gastric ultrasound was small, the sample size of our study was small. Therefore, extrapolation of our results to broader patient groups with various underlying diseases should be performed with caution.

Second, the residual GV was not determined based on the aspiration of actual gastric contents. However, the reproducibility of the GV calculation formula has been proven in previous studies.^[3]^ Moreover, the measurement of GV through gastric suction is not considered to be a reliable tool for assessing the GV.^[2]^ Therefore, we think that GV evaluation determined by gastric suction would not affect our study outcomes.

Third, we compared the 2 different populations; therefore, some differences were found in the demographic characteristics of the patients of the 2 groups. The proportion of female patients in the young group was 100%, whereas that in the old group was 20.1%. However, because sex does not affect the CSA, we believe that the difference in the proportion of female patients would not have affected our outcomes.^[16]^ The differences in weight, body mass index, and ASA class might be because of the differences in sex ratio or age between the groups. However, because these factors do not significantly affect GV assessment, these factors might not have significantly affected our results.^[9]^ Furthermore, comorbid diseases increase with age, and the ASA class was inevitably higher in the elderly group than in young group. The solid fasting time was longer in the elderly group (14 hours) than in the young group (12 hours). However, gastric emptying of liquid is not affected by gastric emptying of solid food because solid food is emptied by peristalsis or pressure pump mechanism.^[25]^ In addition, the fasting time for solids in both the groups exceeded the gastric emptying time of the solid food (5.8 ± 0.8 hours).^[26]^ Therefore, it is thought that 3-hour difference in fasting time for solids between the 2 groups would have not affected our outcomes.

In conclusions, The CSA of the empty stomach greater in elderly patients than in young patients. Different cutoff values of the CSA should be used for predicting pulmonary aspiration risk in old and young patients. To predict high pulmonary aspiration risk in old patients, we suggest that the following CSA cutoff values should be used: CSA supine, 6.92 cm^2^ and CSA RLDP, 10.65 cm^2^.

## Acknowledgments

We would like to thank Editage (www.editage.co.kr) for English language editing. The preprint version of the manuscript is present at https://doi.org/10.21203/rs.3.rs-1137760/v1.

## Author contributions

**Conceptualization:** Kyoung-Ho Ryu, Eunah Cho.

**Data curation:** Jin Hee Ahn, Sinae Kim, Tae-Ryun Gahng, Eunah Cho.

**Formal analysis:** Jin Hee Ahn, Jae-Geum Shim, Mi Yeon Lee, Sinae Kim, Eunah Cho.

**Investigation:** Tae-Ryun Gahng.

**Methodology:** Tae-Ryun Gahng.

**Supervision:** Eunah Cho.

**Writing – original draft:** Jae-Geum Shim, Sung Hyun Lee, Eunah Cho.

**Writing – review & editing:** Sung Hyun Lee, Kyoung-Ho Ryu, Eunah Cho.
